# Spatiotemporal Correlation Analysis for the Incidence of Esophageal and Gastric Cancer From 2010 to 2019: Ecological Study

**DOI:** 10.2196/66655

**Published:** 2025-01-29

**Authors:** Zixuan Cui, Chen Suo, Yidan Zhao, Shuo Wang, Ming Zhao, Ruilin Chen, Linyao Lu, Tiejun Zhang, Xingdong Chen

**Affiliations:** 1 Department of Epidemiology, School of Public Health, Fudan University, Dongan Road 130, Shanghai, 200032, China, 86 15618218427; 2 Shanghai Institute of Infectious Disease and Biosecurity, Shanghai, China; 3 Fudan University Taizhou Institute of Health Sciences, Jiangsu, China; 4 State Key Laboratory of Genetic Engineering, Human Phenome Institute, Zhangjiang Fudan International Innovation Center, Shanghai, China; 5 Yiwu Research Institute of Fudan University, Zhejiang, China; 6 National Clinical Research Center for Aging and Medicine, Huashan Hospital, Shanghai, China

**Keywords:** spatiotemporal analysis, spatiotemporal correlation, esophageal cancer, gastric cancer, cancer, global burden of disease, GBD, average annual percentage change, incidence, epidemiology

## Abstract

**Background:**

Esophageal and gastric cancer were among the top 10 most common cancers worldwide. In addition, sex-specific differences were observed in the incidence. Due to their anatomic proximity, the 2 cancers have both different but also shared risk factors and epidemiological features. Exploring the potential correlated incidence pattern of them, holds significant importance in providing clues in the etiology and preventive strategies.

**Objective:**

This study aims to explore the spatiotemporal correlation between the incidence patterns of esophageal and gastric cancer in 204 countries and territories from 2010 to 2019 so that prevention and control strategies can be more effective.

**Methods:**

The data of esophageal and gastric cancer were sourced from the Global Burden of Disease (GBD). Spatial autocorrelation analysis using Moran I in ArcGIS 10.8 (Esri) was performed to determine spatial clustering of each cancer incidence. We classified different risk areas based on the risk ratio (RR) of the 2 cancers in various countries to the global, and the correlation between their RR was evaluated using Pearson correlation coefficient. Temporal trends were quantified by calculating the average annual percent change (AAPC), and the correlation between the temporal trends of both cancers was evaluated using Pearson correlation coefficients.

**Results:**

In 2019, among 204 countries and territories, the age-standardized incidence rates (ASIR) of esophageal cancer ranged from 0.91 (95% CI 0.65-1.58) to 24.53 (95% CI 18.74-32.51), and the ASIR of gastric cancer ranged from 3.28 (95% CI 2.67-3.91) to 43.70 (95% CI 34.29-55.10). Malawi was identified as the highest risk for esophageal cancer (male RR=3.27; female RR=5.19) and low risk for gastric cancer (male RR=0.21; female RR=0.23) in both sexes. Spatial autocorrelation analysis revealed significant spatial clustering of the incidence for both cancers (Moran I>0.20 and *P*<.001). A positive correlation between the risk of esophageal and gastric cancer was observed in males (*r*=0.25, *P*<.001). The ASIR of both cancers showed a decreasing trend globally. The ASIR for esophageal and gastric cancer showed an AAPC of −1.43 (95% CI −1.58 to −1.27) and −1.76 (95% CI −2.08 to −1.43) in males, and −1.93 (95% CI −2.11 to −1.75) and −1.79 (95% CI −2.13 to −1.46) in females. In addition, a positive correlation between the temporal trends in ASIR for both cancers was observed at the global level across sexes (male *r*=0.98; female *r*=0.98).

**Conclusions:**

Our study shows that there was a significant spatial clustering of the incidence for esophageal and gastric cancer and a positive correlation between the risk of both cancers across countries was observed in males. In addition, a codescending incidence trend between both cancers was observed at the global level.

## Introduction

Globally, cancer is a leading cause of mortality, with its significance continually rising [1-4]. It was estimated that in 2019, cancer accounted for 250 million disability-adjusted life years [3]. Concurrently, according to the Global Cancer Statistics 2020, esophageal and gastric cancer were among the top 10 most common cancers worldwide [5]. In addition, sex-specific differences were observed in the incidence [5]. Due to their anatomic proximity, esophageal and gastric cancer have both different but also a number of shared risk factors and epidemiological features [6-8]. Several published articles have demonstrated that frequent consumption of hot beverages and poor oral hygiene are risk factors for esophageal cancer [9,10], while a high-sodium diet and *Helicobacter pylori* infection are risk factors for gastric cancer [11,12]. Smoking and heavy alcohol consumption are associated with the incidence of both esophageal and gastric cancer [13,14]. There are also some pathological injuries involving both esophagus and stomach, such as gastroesophageal reflux disease and Barrett esophagus, which is related to reflux [15,16]. Furthermore, the high-incidence regions for esophageal cancer are primarily distributed in East Asia, Southeastern Africa, and Northern Europe, while the high-incidence regions for gastric cancer are mainly distributed in East Asia and Eastern Europe [5]. The spatial distribution of these 2 cancers exhibits certain overlapping patterns. However, there has been no global-level spatiotemporal correlation analysis simultaneously examining the incidence of both esophageal and gastric cancer [17-22]. Therefore, systematic exploring the spatiotemporal correlation in the incidence of esophageal and gastric cancer holds significant importance.

Through the spatiotemporal correlation analysis of global incidence of esophageal and gastric cancer, we aim to provide insightful information that would contribute to the prevention and control of these cancers worldwide and the rational allocation of global public health resources.

## Methods

### Data Sources

The Global Burden of Diseases, Injuries, and Risk Factors Study 2019 (GBD 2019), coordinated by the Institute for Health Metrics and Evaluation (IHME) offers comprehensive and comparable data on the epidemiological burden of esophageal and gastric cancer, which includes age-standardized incidence rates (ASIR) recorded annually spanning from 1990 to 2019 [4]. The data of esophageal and gastric cancer were sourced from the Global Health Data Exchange (GHDx) query tool [23]. The GHDx query tool in the GBD 2019 database includes data from 204 countries and territories by sex from 1990 to 2019. Furthermore, according to the geographic location, the world was divided into 21 regions such as East Asia in GBD 2019 database. The quality and integrity of data can be significantly influenced by advancements in medical records and data collection technologies. Therefore, we extracted data on the ASIR (per 100,000 person-years) with 95% uncertainty interval (UI) of esophageal and gastric cancer from 2010 to 2019 due to the higher quality and integrity of recent data in our study. The ASIR was calculated by the direct method. Standardization was crucial in this study as it eliminates the bias when comparing rates. The 95% UI is a range of values that reflects the certainty of an estimate. The sociodemographic index (SDI) is a composite indicator developed by GBD researchers to characterize the developmental status of countries and territories, and it is closely associated with health outcomes. It is the geometric mean of 0 to 1 indices of total fertility rate under the age of 25, mean education for those ages 15 and older, and lag distributed income per capita [24]. The IHME provides publicly available SDI Reference Quintiles and the SDI values for all locations from 1950 to 2020 [24]. Based on the provided SDI Reference Quintiles, all locations can be classified into 5 categories, including low SDI (SDI≤0.46), low-middle SDI (0.46<SDI≤0.61), middle SDI (0.61<SDI≤0.69), high-middle SDI (0.69<SDI≤0.81), and high SDI (SDI>0.81).

### Statistical Analysis

#### Spatial Distribution and Correlation of Esophageal and Gastric Cancer

In the analysis of spatial distribution and correlation of the ASIR of esophageal and gastric cancer, we used ArcGIS 10.8 (Esri) to calculate Global Moran I [25,26], which was used to evaluate whether the incidence of each cancer exhibited spatial clustering. Global Moran I measure spatial autocorrelation based on both feature locations and feature values simultaneously, where the feature here refers to different countries and territories. The tool calculates the Moran I value and both a $z_{I}$-score and *P* value to evaluate the significance of that Index. The Moran I statistic for spatial autocorrelation was given as Multimedia Appendix 1.

Moran I values range between −1 and +1. For the Global Moran I statistic, the null hypothesis states that the attribute being analyzed is randomly distributed among the features in the study area. When the *P* value is statistically significant, we may reject the null hypothesis, and a positive value indicates the spatial distribution of high values and low values in the dataset is more spatially clustered than would be expected if underlying spatial processes were random; a negative value indicates the spatial distribution of high values and low values in the dataset is more spatially dispersed than would be expected if underlying spatial processes were random. A dispersed spatial pattern often reflects some type of competitive process—a feature with a high value repels other features with high values, and it is similar in a feature with a low value. When the *P* value is not statistically significant, we cannot reject the null hypothesis, indicating a random spatial distribution. In spatial autocorrelation analysis, the conceptualization of spatial relationships adheres to the Inverse_Distance rule.

In addition, by comparing the ASIR levels of esophageal and gastric cancer in each country and territory against those of the global ASIR levels, we obtained the corresponding risk ratio (RR) to quantify the level of relative risk. Countries and territories with an RR value≤0.50 were classified as low-risk area, while an 0.50<RR value<2.00 and RR value≥2.00 were classified as medium-risk area and high-risk area, respectively. We then used the Pearson correlation coefficient to evaluate the correlation between the RR of esophageal and gastric cancer in both sex groups. We also evaluated the relationship between SDI quintiles and the distribution of different risk areas.

#### Temporal Trend and Correlation of Esophageal and Gastric Cancer

In the analysis of temporal trends and correlation of the ASIR of esophageal and gastric cancer, we used the National Cancer Institute Joinpoint Regression Program (version 5.0.2) to calculate the average annual percent change (AAPC) of the ASIR for esophageal and gastric cancer across 204 countries and territories during 2010‐2019. The CI was set at 95%. AAPC is a statistical indicator used to describe the average annual change rate of the ASIR over a certain period. It provides a comprehensive assessment of the overall trend of ASIR over the entire study period by weighting the annual percentage change over multiple time periods. If both the estimation of AAPC and its lower boundary of 95% CI were >0, the ASIR was considered to be in an increasing trend; if both the estimation of AAPC and its upper boundary of 95% CI were <0, the ASIR was considered to be in a decreasing trend. Otherwise, the ASIR was considered to be stable over time. In the correlation analysis, the Pearson correlation coefficient was used to evaluate the correlation between the temporal trends in the ASIR of esophageal and gastric cancer. A Pearson correlation coefficient >0 with a *P* value less than the specified significance level indicated a significant positive correlation, while a coefficient <0 with a *P* value less than the specified significance level indicated a significant negative correlation. In addition, we assessed the correlation between the temporal trends in the ASIR of both cancers across SDI quintiles.

Given the significant sex differences in the incidence of esophageal and gastric cancer [27,28] and the lifestyle disparities, all analyses were stratified by sex. ArcGIS (version 10.8) and Joinpoint Regression Program (version 5.0.2) were used for spatial autocorrelation analysis and temporal trend analysis of ASIR respectively. All data analyses were conducted in software R (version 4.3.2; R Foundation for Statistical Computing) and R Studio (Posit). A *P* value of less than .05 was considered statistically significant.

### Ethical Considerations

This study did not involve human participants and animals. Ethics approval was not applicable for this study, as this study used existing good quality data that were aggregated at the population level. Data available for download on IHME websites are publicly available and can be used, shared, modified or built upon by noncommercial users in accordance with the IHME Free-of-Charge Non-Commercial User Agreement [29].

## Results

### Spatial Distribution and Correlation Between the Esophageal and Gastric Cancer Incidence in 2019

**Figure 1.**
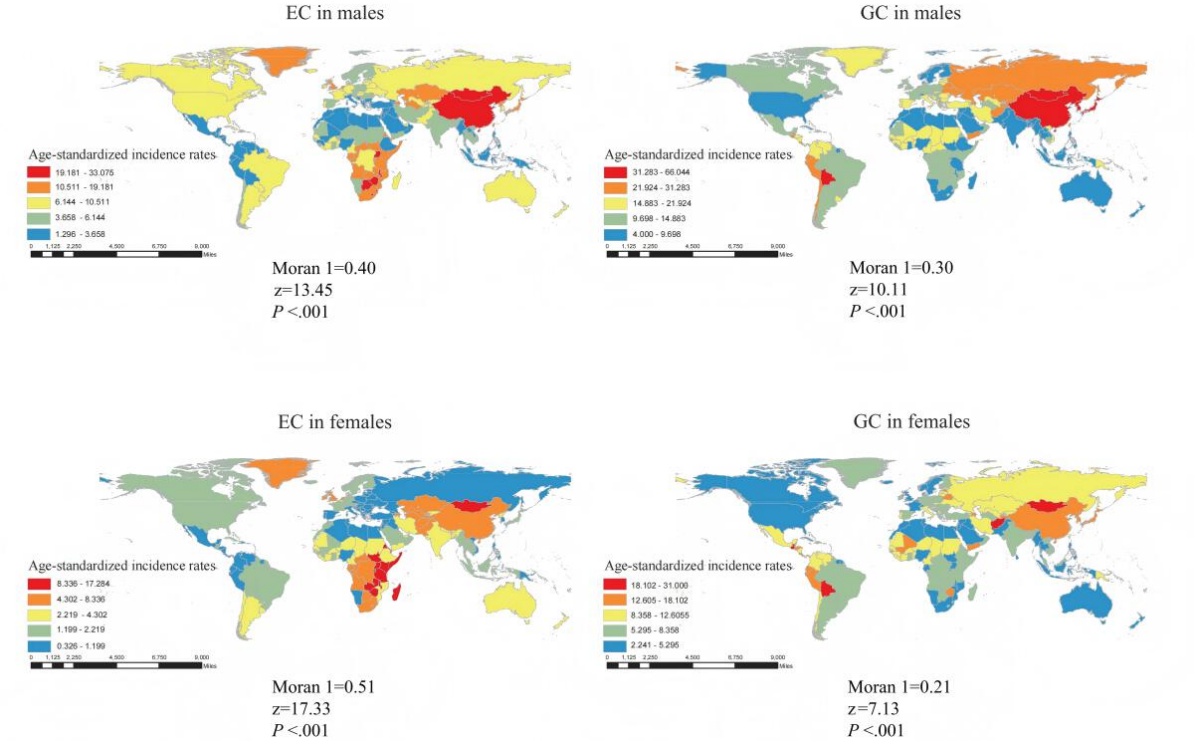
ASIR of esophageal cancer (EC) and gastric cancer (GC) in 204 countries and territories in 2019, by sex. EC: esophageal cancer; GC: gastric cancer; ASIR: age-standardized incidence rate.

The study included data on the incidence of esophageal and gastric cancer from 204 countries and territories and 21 geographic regions. In 2019, the global ASIR of esophageal and gastric cancer were 6.51 (95% CI 5.69-7.25) and 15.59 (95% CI 14.11-17.15) per 100,000 person-years, respectively, with considerable variation observed across regions and sex groups. Among the 204 countries and territories provided by the GBD dataset, the ASIR of esophageal cancer ranged from a minimum of 0.91 (95% CI 0.65-1.58) in Nigeria to a maximum of 24.53 (95% CI 18.74-32.51) in Malawi, while the ASIR of gastric cancer ranged from a minimum of 3.28 (95% CI 2.67-3.91) in Malawi to a maximum of 43.70 (95% CI 34.29-55.10) in Mongolia. Overall, the ASIR of esophageal and gastric cancer was significantly higher in males than in females, and within the sex group, the ASIR of gastric cancer was significantly higher than that of esophageal cancer at the global level (Figure 1). In 2019, Malawi had the highest ASIR of esophageal cancer in both sexes (male: 33.08, 95% CI 24.44-44.43; female: 17.28, 95% CI 12.64-23.50), and Mongolia was ranked the top 5 for both esophageal (male: 30.48, 95% CI 22.58-37.90; female: 16.03, 95% CI 7.50-21.37) and gastric cancer (male: 66.04, 95% CI 51.50-82.68; female: 28.18, 95% CI 21.53-36.56) in both sexes (Table S1 in Multimedia Appendix 2). In a broader scale, among males, the ASIR of esophageal and gastric cancer in East Asia, Central Asia and high-income Asia Pacific all ranked within the top 6 among 21 geographic regions. Among males, East Asia had the highest ASIR for both esophageal (21.70, 95% CI 16.37-26.61) and gastric cancer (46.67, 95% CI 37.63-56.82), while High-income Asia Pacific ranked fifth for esophageal cancer (10.68, 95% CI 8.87-12.89) and second for gastric cancer (41.91, 95% CI 35.58-49.40), and Central Asia ranked sixth (9.51, 95% CI 8.37-11.53) and fifth (24.08, 95% CI 21.82-26.57), respectively. Similarly, in females, the regions with the top 6 ASIR for esophageal and gastric cancer were East Asia and Central Asia. East Asia ranked second for esophageal cancer (6.67, 95% CI 4.39-8.38) and third for gastric cancer (15.65, 95% CI 12.80-19.05), while Central Asia ranked fifth (4.70, 95% CI 4.19-5.29) and sixth (10.76, 95% CI 9.81-11.87), respectively (Figures S1 and S2 in Multimedia Appendix 2). The spatial autocorrelation analysis also showed that there was a significant clustering phenomenon in the spatial distribution of the incidence of esophageal and gastric cancer in both sex groups (all Moran I>0.20 and *P* value <.001; Figure 1).

**Figure 2.**
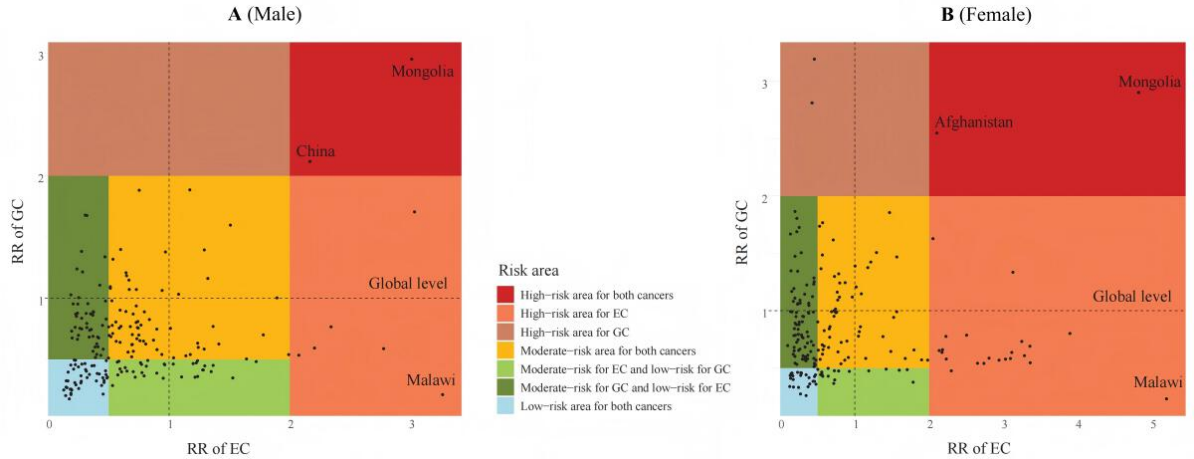
Risk area classification map of esophageal cancer (EC) and GC (gastric cancer) for 204 countries and territories in 2019, by sex. A. Risk area classification map of EC and GC for 204 countries and territories in males. B. Risk area classification map of EC and GC for 204 countries and territories in females. EC: esophageal cancer; GC: gastric cancer; ASIR: age-standardized incidence rates; RR: risk ratio; obtained by comparing the ASIR levels of esophageal and gastric cancer in each country and territory against those of the global ASIR levels.

The 2019 risk area classification results were shown in Figure 2. In 2019, China and Mongolia belonged to the high-risk area for both esophageal and gastric cancer in males (Figure 2), while Afghanistan and Mongolia belonged to the high-risk area for both cancers in the females (Figure 2). Mongolia was identified as an overlapped high-risk area for both esophageal cancer and gastric cancer in males and females. In addition, the number of countries and territories belonging to the high-risk area for esophageal cancer alone was significantly higher in females than in males, and the number of countries and territories belonging to the low-risk area for both esophageal and gastric cancer was much lower in females than in males. Furthermore, no country or territory in males belonged to the high-risk area for gastric cancer alone, while in females, there were 2 countries (Guatemala and Bolivia). It is noteworthy that Malawi had the highest RR value for esophageal cancer (male RR=3.27; female RR=5.19) and a low RR value for gastric cancer (male RR=0.21; female RR=0.23) in both males and females. We further conducted Pearson correlation analysis for the RR values of esophageal and gastric cancer, and found that there was a positive correlation between the RR values of esophageal and gastric cancer in males (*r*=0.25; *P*<.001), but no significant correlation was found in females (*r*=0.02; *P*=.77).

### Temporal Trend and Correlation Between Esophageal and Gastric Cancer Incidence From 2010 to 2019

From 2010 to 2019, at the global level, the ASIR of esophageal cancer ranged from a peak of 7.50 (95% CI 6.10-8.02) in 2010 to a lowest point of 6.44 (95% CI 5.79-7.00) in 2017, while the ASIR of gastric cancer ranged from a peak of 18.25 (95% CI 17.14-19.22) in 2010 to a lowest point of 15.59 (95% CI 14.11-17.15) in 2019. And the ASIR of esophageal and gastric cancer decreased significantly in both sex groups globally. The AAPC for esophageal and gastric cancer in males was −1.43 (95% CI −1.58 to −1.27) and −1.76 (95% CI −2.08 to −1.43), respectively, while in females, the AAPC for esophageal and gastric cancer was −1.93 (95% CI −2.11 to −1.75) and −1.79 (95% CI −2.13 to −1.46), respectively. We selected all the high-risk countries and territories which had the top 5 ASIR of esophageal or gastric cancer in 2019 in both sex groups (14 countries and territories were finally presented in the table due to partial overlap; Table S2 in Multimedia Appendix 3). In males, the ASIR for esophageal and gastric cancer both showed a decreasing trend in 10 countries and territories, while in females, this trend was observed in 11 countries and territories. Furthermore, in all countries and territories except Bolivia, there was a strong positive correlation between the temporal trends in the ASIR of esophageal and gastric cancer (Pearson correlation coefficient>0.5). In addition, it is noteworthy that despite Cabo Verde being among the top 5 countries for ASIR of esophageal and gastric cancer in males, the ASIR for these cancers continued to show a significant increasing trend in males, with an increasing trend also observed in females.

**Figure 3.**
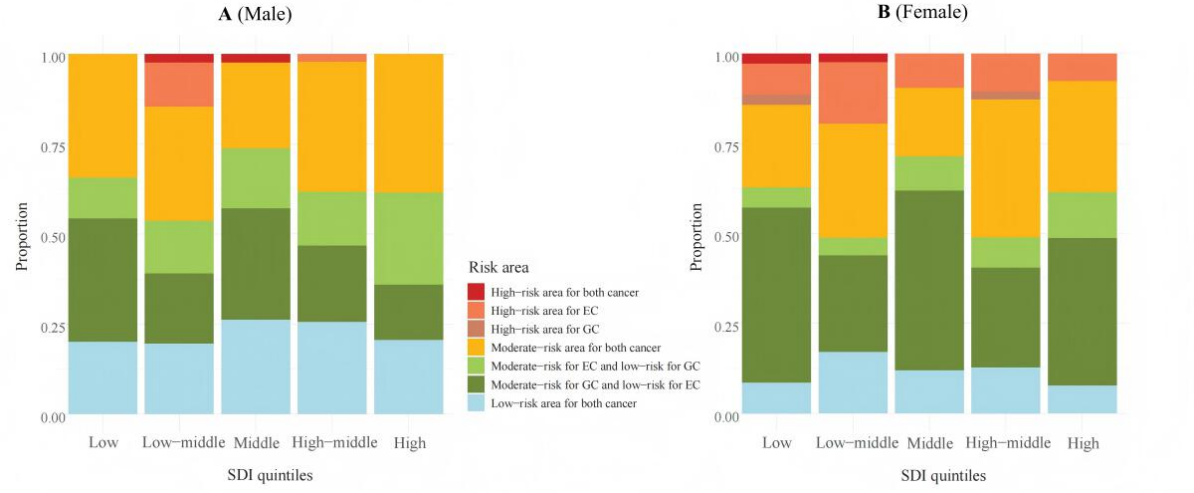
Stacked bar plot of sociodemographic index (SDI) quintiles and different risk areas, by sex. (A) The stacked bar plot of SDI quintiles and different risk areas in males. (B) The stacked bar plot of SDI quintiles and different risk areas in females. EC: esophageal cancer; GC: gastric cancer; SDI: sociodemographic index.

We also performed Pearson correlation analyses for the temporal trends between esophageal and gastric cancer ASIR at the global level and across all 204 countries and territories. At the global level, the Pearson correlation coefficient (*r*) for the temporal trends in the ASIR for both cancers were 0.98 in males and 0.98 in females. The results also showed that a positive correlation between temporal trends in the ASIR of the 2 cancers was observed in most countries and territories, regardless of sex (Figure 3 in Multimedia Appendix 3). A normality test on the distribution of Pearson correlation coefficients across 204 countries and territories for both males and females indicated that the distributions were not normal. Wilcoxon rank-sum test results showed that there were differences (*P*<.001) in the distribution of Pearson correlation coefficient data between males and females in these 204 countries and territories, and the median Pearson correlation coefficient was higher for females than for males.

### The Correlation Between SDI and Both Risk Areas and Temporal Trend

Based on the SDI Reference Quintiles publicly provided by the IHME and the SDI values for all locations since 1950 [24], we collected the SDI for 204 countries and territories in 2019, and classified these countries and territories into different SDI quintiles according to the SDI Reference Quintiles. The composition of risk areas for different SDI quintiles was shown in Figure 3. As shown in Figure 3, the high-risk area for both cancers all distributed in the middle and below SDI quintiles. In males, high-risk areas were distributed only in the low-middle, middle, and high-middle SDI quintiles (Figure 3), while high-risk areas were distributed across all SDI quintiles in females (Figure 3). The chi-square test results show that there was no difference in the composition ratio of risk areas among various SDI quintiles in both sex groups.

As shown in Table 1, the ASIR of esophageal and gastric cancer both showed a downward trend in all SDI quintiles from 2010 to 2019, with all 95% CI excluding 0. In both sexes, the average annual percentage decrease in ASIR for esophageal and gastric cancer in the middle SDI quintile was both substantial. In addition, the decrease in ASIR for esophageal cancer in this quintile over the past decade was significantly more substantial than in other SDI quintiles. In the middle SDI quintile, the average annual percentage decrease in ASIR for esophageal cancer over the decade was more substantial than that for gastric cancer across both sexes. Conversely, in other SDI quintiles except for females in the low-middle SDI, the decrease in ASIR for esophageal cancer was less substantial than that for gastric cancer. Pearson correlation analysis indicated that the correlation between the temporal trends in the ASIR for esophageal and gastric cancer was high in all SDI quintiles (Table 1 and Figure S4 in Multimedia Appendix 4). Since the distributions of Pearson correlation coefficients in each SDI quintile was nonnormal in both sex groups after normality test, the Kruskal-Wallis test was used to assess the differences in correlation coefficients among these SDI quintiles. The Kruskal-Wallis test indicated no significant differences in the correlation between the temporal trends in the ASIR of esophageal and gastric cancer across various SDI quintiles in both sex groups.

**Table 1.** Average annual percent change (AAPC) in age-standardized incidence rates (ASIR) of esophageal cancer (EC) and gastric cancer (GC) among different sociodemographic index (SDI) quintiles, 2010‐2019 (AAPC, %).

Location	Male (95% CI)	*r* ^a^	Female (95% CI)	*r* ^a^
**Low SDI**		0.85		0.94
EC	−0.27^b^ (−0.42 to −0.12)		−0.39^b^ (−0.46 to −0.33)	
GC	−1.37^b^ (−1.53 to −1.21)		−0.74^b^ (−0.84 to −0.65)	
**Low−middle SDI**		0.88		0.95
EC	−0.21^b^ (−0.34 to −0.09)		−0.43^b^ (−0.74 to −0.12)	
GC	−1.19^b^ (−1.41 to −0.98)		−0.67^b^ (−0.82 to −0.52)	
**Middle SDI**		0.87		0.94
EC	−2.56^b^ (−2.77 to −2.35)		−3.79^b^ (−4.04 to −3.53)	
GC	−1.90^b^ (−2.13 to −1.67)		−2.26^b^ (−2.64 to −1.88)	
**High−middle SDI**		0.76		0.94
EC	−0.95^b^ (−1.28 to −0.62)		−1.58^b^ (−1.84 to −1.31)	
GC	−1.66^b^ (−1.84 to −1.48)		−2.13^b^ (−2.28 to −1.97)	
**High SDI**		0.88		0.95
EC	−0.92^b^ (−1.07 to −0.76)		−0.79^b^ (−0.88 to −0.69)	
GC	−2.46^b^ (−2.68 to −2.23)		−1.94^b^ (−2.31 to −1.56)	

a Median Pearson correlation coefficients between temporal trends in the ASIR of esophageal and gastric cancer for countries and territories in each SDI quintile from 2010 to 2019.

b Indicates that 95% CI did not include 0.

## Discussion

### Principal Results

This study is the first to simultaneously report on the global temporal trends and spatial distribution of the ASIR for esophageal and gastric cancer, as well as their correlation based on GBD 2019 data. Our results show that there was a significant spatial clustering of the incidence for both cancers and a positive correlation between the risk of esophageal and gastric cancer across countries was observed in males. In addition, a codescending incidence trend between both cancers was observed in most countries and territories.

Due to the significant differences in the incidence of esophageal and gastric cancer between males and females [27,28], and the disparities in lifestyle, sex stratification was conducted in all analysis. In consistent with the previous studies, the risk of both esophageal and gastric cancer was high in Asia (especially East Asia and Central Asia) [5,30,31]. In addition, among Asian countries, the incidence of esophageal and gastric cancer was particularly serious in Mongolia (in 2019, the ASIR of both esophageal and gastric cancer ranked in the top 5 among both sexes). For Mongolia, the high incidence of esophageal cancer may be associated with high levels of fluoride in drinking water or drinking hot milk tea [32]. In addition, local cooking and heating mainly rely on coal and wood, resulting in high levels of fine particulate matter (diameter<2.5 μm), may also be one of the main reasons for the high incidence of esophageal cancer [33]. The spatial clustering phenomenon and the variation of geographical distribution in ASIR of esophageal and gastric cancer may be due to different lifestyles and environmental factors caused by differences in geographical and socioeconomic factors, as well as different histological subtypes of both cancers [34]. Compared with gastric cancer, there were more countries and territories with extremely high ASIR for esophageal cancer relative to the global level, which may be the reason why there were more countries and territories classified as the high-risk area for esophageal cancer than gastric cancer in this study. Although females were generally considered to have a lower risk of esophageal and gastric cancer than males, there were more countries and territories where females were at extremely high risk of esophageal and gastric cancer relative to the global level. Those countries and territories, where the risk of esophageal and gastric cancer was much higher than the global level should be taken seriously. In addition, it is noteworthy that Malawi exhibited the highest risk for esophageal cancer alongside a very low risk for gastric cancer in both sex groups. The previous studies found that African countries with a higher incidence of esophageal cancer tend to have a lower estimated supply of selenium in their diets [35]. Therefore, the generally low selenium intake of the population in Malawi mainly caused by the reduced soil-to-crop selenium transfers in the local typical low pH soils may be one of the reasons for this phenomenon [35-37]. Since Malawi is one of the major tobacco producers in Africa, smokers have easier access to self-rolled tobacco (without filters), which will lead to smokers of this form of tobacco receive a higher dose of the carcinogenic products within the tobacco in comparison to store-bought cigarettes, thereby increasing the risk of developing esophageal cancer [38]. In addition, it has been reported that mycotoxins such as fumonisin B-1 stored in grain are fairly common in maize samples from Malawi, and although a direct causal relationship has not been established, this may be one of the reasons for the high incidence of esophageal cancer there [38]. The main source of energy in Malawian households is wood burning, which produces incomplete combustion products such as polycyclic aromatic hydrocarbons and may also be responsible for the high incidence of esophageal cancer [39]. In Pearson correlation analysis on the RR values for esophageal and gastric cancer among males and females, a positive correlation between the risk of incidence for esophageal and gastric cancer was observed in males, whereas no such correlation was found in females. Taking into account the possibility that the results may be influenced by a greater number of extreme values in females, we conducted a correlation analysis after removing some outliers. Nevertheless, no statistically significant correlation was found. One possible explanation is that smoking, as a primary common risk factor for both esophageal and gastric cancer, occurs at a significantly higher prevalence in males than in females (about 4‐5 times higher) [40].

From 2010 to 2019, there was a global trend of decline in the ASIR for both esophageal and gastric cancer among males and females. The decline in the incidence of both cancers may be attributed to the improvements in the socioeconomic level and population health awareness in recent years [41-43]. It is worth mentioning that there were still some countries and territories, even some high-risk countries and territories including Cabo Verde, exhibited an increasing trend in the ASIR of esophageal and gastric cancer from 2010 to 2019. Although the significant increase of cancer registers compared with the past may be one of the reasons, it is necessary to actively control the incidence of esophageal and gastric cancer for those countries and territories showing an increasing trend. The reason for the stronger positive correlation between the temporal trends of esophageal and gastric cancer in females compared to that in males is unclear. However, it is certain that a significant positive correlation exists, which may be related to the shared risk factors due to their anatomic proximity.

When exploring the correlation between SDI and risk areas classified by RR values of esophageal and gastric cancer, no statistically significant difference was found in the composition ratio of risk areas across various SDI quintiles. This may be related to the low number of countries and territories belonging to the high-risk area for both cancers and gastric cancer alone. Consistent with the global trend, all SDI quintiles exhibited a decreasing trend in the ASIR of esophageal and gastric cancer, which indicated that, overall, the incidence of esophageal and gastric cancer had been improving in regions with different SDI quintiles from 2010 to 2019. Compared with other quintiles, the ASIR of esophageal and gastric cancer in middle SDI quintile both showed a substantial decreasing trend, which may be related to the high ASIR of both esophageal and gastric cancer in middle SDI quintile. Although the ASIR of gastric cancer was significantly higher than that of esophageal cancer, in contrast to other SDI quintiles, the decreasing trend in ASIR for esophageal cancer over the decade was more substantial than that for gastric cancer in middle SDI quintile. This opposite phenomenon, observed in the middle SDI quintile, calls for more rational public policies to strengthen the control of gastric cancer related risk factors. The correlation between the temporal trends in the incidence of esophageal and gastric cancer was strong among different SDI quintiles, and no statistically significant difference of this correlation was found across various SDI quintiles.

### Strengths and Limitations

Our study has numerous strengths, including the ability to visually display the risk of both esophageal and gastric cancer in various countries and territories through risk area classification. This aids in developing prevention strategies tailored to shared or distinct risk factors of the 2 digestive cancers, while also considering local characteristics. Our study also has some limitations. First, the data we analyzed is sourced from the GBD 2019, the quality of some data, especially that from low-income or low SDI countries, is difficult to be guaranteed. Second, esophageal cancer includes 2 histological subtypes—esophageal squamous cell carcinoma and esophageal adenocarcinoma [44,45], while gastric cancer includes cardia gastric cancer and noncardia gastric cancer subtypes [46,47]. There are analogous and distinct etiologies with modifiable risk factors between these subtypes, as well as epidemiological characteristics [48-51]. However, due to the limitations of the GBD database, our study did not differentiate the subtypes of esophageal cancer and gastric cancer. Third, the results of Moran I are highly dependent on the choice of the conceptualization of spatial relationships. For global analysis, selecting an appropriate conceptualization is challenging, especially when regions are separated by natural barriers such as oceans. Contiguity measure may fail to capture spatial relationships across such barriers, while distance measure requires careful consideration of distance thresholds. In addition, Moran I is typically calculated for a single time point, which does not account for dynamic changes in spatial patterns over time. Furthermore, more detailed work is required to identify the primary modifiable risk factors in high-risk areas for each cancer, which will aid in more precisely reducing the global incidence of esophageal and gastric cancer.

### Conclusions

This spatiotemporal correlation study simultaneously investigates esophageal cancer and gastric cancer, which share many risk factors. The results shows that there was a significant spatial clustering of the incidence for esophageal and gastric cancer and a positive correlation between the risk of both cancers across countries was observed in males. In addition, a codescending incidence trend between both cancers was observed at the global level. Despite the overall declining trend in the incidence rates of both esophageal and gastric cancer, they still pose a heavy disease burden worldwide. Analyzing the correlations in the global distribution and temporal trends of the incidence of esophageal and gastric cancer can help to gain a deeper understanding of the homogeneity and heterogeneity in the incidence pattern of these 2 cancers to optimize the allocation of global public health resources.

## Supplementary material

10.2196/66655 Multimedia Appendix 1 Formulas for calculating the Moran I.

10.2196/66655 Multimedia Appendix 2 The age-standardized incidence rates (ASIR) of both cancers in 2019.

10.2196/66655 Multimedia Appendix 3 Average annual percent change (AAPC) in age-standardized incidence rates (ASIR) of both cancers and the correlation of temporal trends.

10.2196/66655 Multimedia Appendix 4 Correlation of temporal trends in different sociodemocratic index (SDI) quintiles.

## Abbreviations

AAPC: average annual percent change
ASIR: age-standardized incidence rates
GBD: Global Burden of Disease
GHDx: Global Health Data Exchange
IHME: Institute for Health Metrics and Evaluation
RR: risk ratio
SDI: socio-demographic index
UI: uncertainty interval
