# Supplementary material for: Spatiotemporal Correlation Analysis for the Incidence of Esophageal and Gastric Cancer From 2010 to 2019: Ecological Study
Source: JMIR Cancer. 2025 Jan 29;11:e66655. doi: 10.2196/66655 (PMC11798535; doi:10.2196/66655)
Supplement: Multimedia Appendix 1 [file cancer-v11-e66655-s001.docx]

Multimedia Appendix 1: Formulas for calculating the Moran's I.

Spatiotemporal correlation analysis in the incidence of esophageal and gastric cancer from 2010 to 2019: Longitudinal Observational Study

The Moran's I statistic for spatial autocorrelation is given as:

$$I=\frac{n}{S_{0}}\frac{\sum_{i=1}^{n} \sum_{j=1}^{n} w_{i,j}z_{i}z_{j}}{\sum_{i=1}^{n} z_{i}^{2}}$$

where $z_{i}$ is the deviation of an attribute for feature $i$ from its mean $(x_{i}-\bar{X})$, $w_{i,j}$ is the spatial weight between feature $i$ and $j$, $n$ is equal to the total number of features, and $S_{0}$ is the aggregate of all the spatial weights:

$$S_{0}=\sum_{i=1}^{n} \sum_{j=1}^{n} w_{i,j}$$

The $z_{I}$-score for the statistic is computed as:

$$z_{I}=\frac{I-E\left[ I \right]}{\sqrt{V\left[ I \right]}}$$

where:

$$E\left[ I \right]={-1}/\left( n-1 \right)$$

$$V\left[ I \right]=E\left[ I^{2} \right]-E\left[ I \right]^{2}$$
