# Supplementary material for: Spatiotemporal Correlation Analysis for the Incidence of Esophageal and Gastric Cancer From 2010 to 2019: Ecological Study
Source: JMIR Cancer. 2025 Jan 29;11:e66655. doi: 10.2196/66655 (PMC11798535; doi:10.2196/66655)
Supplement: Multimedia Appendix 2 [file cancer-v11-e66655-s002.docx]

Multimedia Appendix 2: The ASIR of both cancers in 2019.

Spatiotemporal correlation analysis in the incidence of esophageal and gastric cancer from 2010 to 2019: Longitudinal Observational Study

Table of content

Figure S1. The ASIR of EC in 21 geographic regions in 2019, by sex.

Figure S2. The ASIR of GC in 21 geographic regions in 2019, by sex.

Table S1. Countries and territories with top five ASIR of EC or GC in 2019, by sex.





Figure S1. The ASIR of EC in 21 geographic regions in 2019, by sex. (ASIR: age-standardized incidence rates; EC: esophageal cancer)





Figure S2. The ASIR of GC in 21 geographic regions in 2019, by sex. (ASIR: age-standardized incidence rates; GC: gastric cancer)

Table S1. Countries and territories with top five ASIR of EC or GC in 2019, by sex

| EC^m^ | | GC^m^ | |  | EC^f^ | | GC^f^ | | |
| --- | --- | --- | --- | --- | --- | --- | --- | --- | --- |
| Location | ASIR (95%UI) | Location | ASIR (95%UI) |  | Location | ASIR (95%UI) | | Location | ASIR (95%UI) |
| Malawi | 33.08 (24.44, 44.43) | Mongolia | 66.04 (51.50, 82.68) |  | Malawi | 17.28 (12.64, 23.50) | | Bolivia | 31.00 (23.40, 38.77 |
| Cabo Verde | 30.72 (25.05, 36.27) | China | 47.35 (38.00, 57.95) |  | Mongolia | 16.03 (7.50, 21.37) | | Mongolia | 28.18 (21.53, 36.56) |
| Mongolia | 30.48 (22.58, 37.90) | Japan | 42.16 (34.10, 51.58) |  | Eritrea | 12.96 (7.73, 18.33) | | Guatemala | 27.29 (21.84, 33.71) |
| Eswatini | 28.13 (17.93, 37.74) | South Korea | 42.09 (34.61, 50.52 |  | Uganda | 11.18 (8.48, 13.87) | | Afghanistan | 24.74 (15.65, 32.95) |
| Lesotho | 23.72 (15.36, 30.46) | Cabo Verde | 38.15 (32.62, 44.11) |  | Kenya | 11.17 (7.99, 17.83) | | South Korea | 18.10 (14.89, 21.59) |

EC – esophageal cancer, GC – gastric cancer, ASIR – age-standardized incidence rates, UI – uncertainty interval. ^m^ the ASIR of esophageal or gastric cancer in males. ^f^ the ASIR of esophageal or gastric cancer in females
