# Supplementary material for: Spatiotemporal Correlation Analysis for the Incidence of Esophageal and Gastric Cancer From 2010 to 2019: Ecological Study
Source: JMIR Cancer. 2025 Jan 29;11:e66655. doi: 10.2196/66655 (PMC11798535; doi:10.2196/66655)
Supplement: Multimedia Appendix 3 [file cancer-v11-e66655-s003.docx]

Multimedia Appendix 3: AAPC in ASIR of both cancers and the correlation of temporal trends.

Spatiotemporal correlation analysis in the incidence of esophageal and gastric cancer from 2010 to 2019: Longitudinal Observational Study

Table of content

Table S2. AAPC in ASIR of EC and GC among 14 high-risk countries and territories, 2010-2019 (AAPC, %)

Figure S3. The histogram and boxplot of Pearson correlation coefficient between temporal trends in the ASIR of EC and GC in 204 countries and territories from 2010 to 2019, by sex. ASIR – age-standardized incidence rates, EC – esophageal cancer, GC – gastric cancer.

Table S2. AAPC in ASIR of EC and GC among 14 high-risk countries and territories^a^, 2010-2019 (AAPC, %)

| Location | Male | | |  | Female | | |
| --- | --- | --- | --- | --- | --- | --- | --- |
|  | EC (95%CI) | GC (95%CI) | r^b^ |  | EC (95%CI) | GC (95%CI) | r^b^ |
| Global | −1.43^d^ (−1.58-−1.27) | −1.76^d^ (−2.08-−1.43) | 0.98 |  | −1.93^d^ (−2.11-−1.75) | −1.79^d^ (−2.13-−1.46) | 0.98 |
| Afghanistan | −0.43^d^ (−0.55-−0.30) | −1.24^d^ (−1.32-−1.16) | 0.97 |  | −0.68^d^ (−0.76-−0.60) | −0.86^d^ (−0.95-−0.7) | 0.998 |
| Bolivia | 0.74^d^ (0.44-1.04) | −0.004 (−0.17-0.16) | 0.46 |  | −0.29^d^ (−0.41-−0.17) | −0.61^d^ (−0.62-−0.59) | 0.90 |
| Cabo Verde | 5.11^d^ (1.39-8.95) | 4.88^d^ (0.69-9.24) | 0.997 |  | 2.38^d^ (1.59-3.17) | 2.27^d^ (1.60-2.95) | 0.99 |
| China | −2.35^d^ (−2.57-−2.12) | −1.84^d^ (−2.08-−1.61) | 0.99 |  | −4.00^d^ (−4.32-−3.68) | −2.75^d^ (−3.42-−2.06) | 0.996 |
| Eritrea | −1.37^d^ (−1.52-−1.22) | −1.67^d^ (−1.75-−1.60) | 0.9997 |  | −0.44^d^ (−0.58-−0.30) | −0.36^d^ (−0.48-−0.25) | 0.997 |
| Eswatini | −1.68^d^ (−1.77-−1.60) | −2.20^d^ (−2.58-−1.81) | 0.99 |  | −2.52^d^ (−2.62-−2.41) | −2.60^d^ (−2.84-−2.35) | 0.9998 |
| Guatemala | −1.37^d^ (−1.76-−0.97) | −2.96^d^ (−3.46-−2.45) | 0.96 |  | −1.40^d^ (−1.67-−1.14) | −2.79^d^ (−3.21-−2.36) | 0.99 |
| Japan | −2.01^d^ (−2.19-−1.83) | −3.38^d^ (−3.60-−3.16) | 0.998 |  | −0.63^d^ (−0.85-−0.42) | −2.98^d^ (−3.43-−2.52) | 0.94 |
| Kenya | −1.10^d^ (−1.25-−0.94) | −2.16^d^ (−2.29-−2.04) | 0.97 |  | −0.08 (−0.18-0.02) | −0.47^d^ (−0.54-−0.41) | 0.65 |
| Lesotho | −0.14 (−0.35-0.06) | −0.87^d^ (−1.03-−0.70) | 0.77 |  | −0.81^d^ (−1.29-−0.34) | −0.86^d^ (−1.27-−0.46) | 0.999 |
| Malawi | −1.23^d^ (−1.48-−0.98) | −2.04^d^ (−2.23-−1.86) | 0.997 |  | −0.48^d^ (−0.53-−0.42) | −0.86^d^ (−0.97-−0.75) | 0.98 |
| Mongolia | −0.34 (−0.74-0.05) | −1.00^d^ (−1.45-−0.56) | 0.91 |  | −2.11^d^ (−2.37-−1.84) | −2.14^d^ (−2.48-−1.80) | 0.999 |
| South Korea | −1.18^d^ (−1.84-−0.52) | −4.65^d^ (−5.14-−4.15) | 0.99 |  | −0.62^d^ (−1.04-−0.21) | −2.78^d^ (−3.22-−2.34) | 0.86 |
| Uganda | −0.75^d^ (−1.06-−0.43) | −1.76^d^ (−1.89-−1.63) | 0.95 |  | 0.13 (−0.23-0.50) | −0.20 (−0.48-0.09) | 0.53 |

^d^ Indicates that 95%CI did not include 0.  ^a^ high-risk countries and territories are those that ranked in the top five for esophageal or gastric cancer among males and females in 2019. ^b^ Pearson correlation coefficient between temporal trends in the ASIR of esophageal and gastric cancer from 2010 to 2019. EC – esophageal cancer, GC – gastric cancer, ASIR – age-standardized incidence rates, CI – confidence interval, AAPC – average annual percent change.


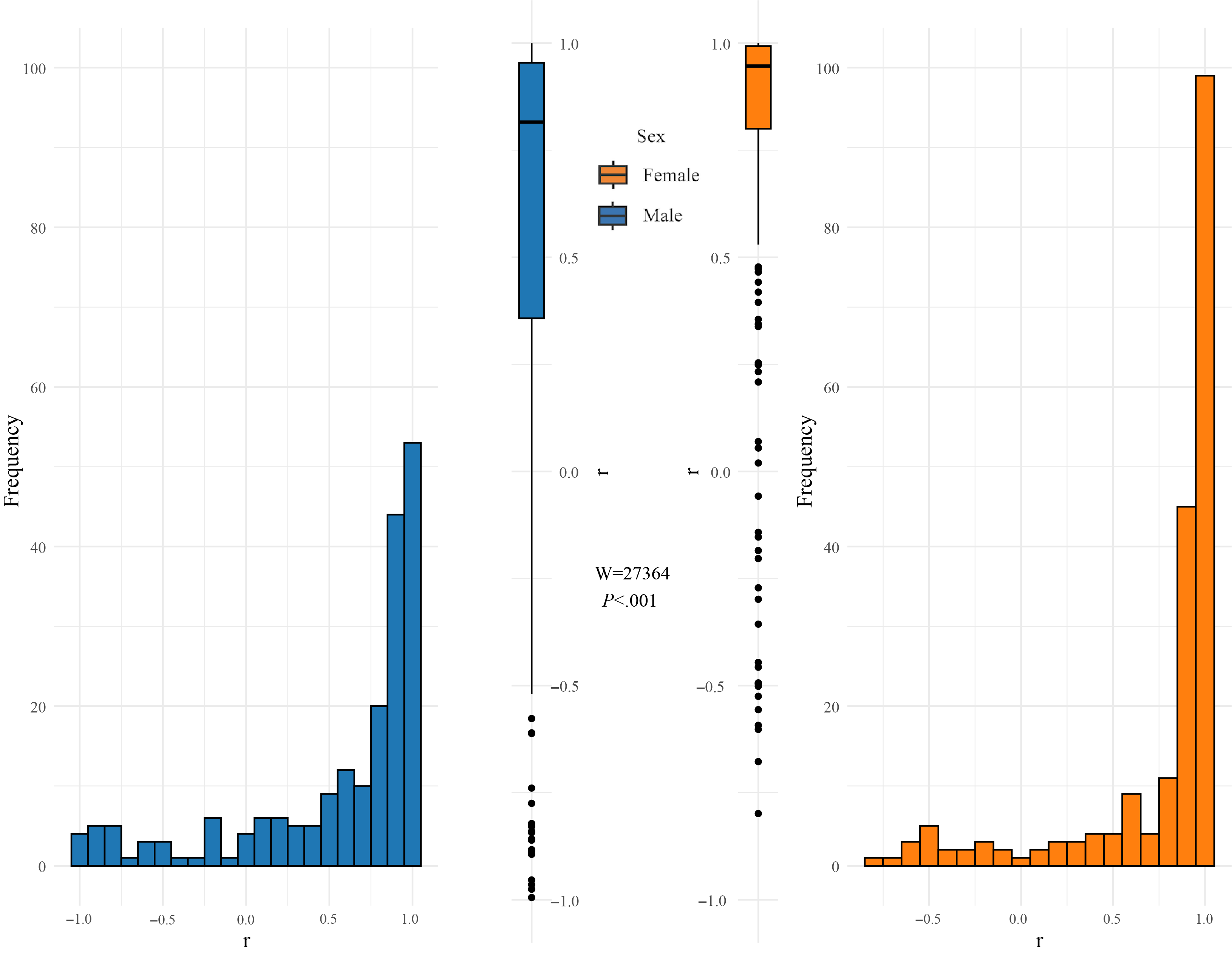


Figure S3. The histogram and boxplot of Pearson correlation coefficient between temporal trends in the ASIR of EC and GC in 204 countries and territories from 2010 to 2019, by sex. ASIR – age-standardized incidence rates, EC – esophageal cancer, GC – gastric cancer.
