# Supplementary material for: Spatiotemporal Correlation Analysis for the Incidence of Esophageal and Gastric Cancer From 2010 to 2019: Ecological Study
Source: JMIR Cancer. 2025 Jan 29;11:e66655. doi: 10.2196/66655 (PMC11798535; doi:10.2196/66655)
Supplement: Multimedia Appendix 4 [file cancer-v11-e66655-s004.docx]

Multimedia Appendix 4: Correlation of temporal trends in different SDI quintiles.

Spatiotemporal correlation analysis in the incidence of esophageal and gastric cancer from 2010 to 2019: Longitudinal Observational Study





Figure S4. Boxplot of Pearson correlation coefficient between temporal trends in the ASIR of EC and GC for different SDI quintiles from 2010 to 2019, by sex. ASIR – age-standardized incidence rates, EC – esophageal cancer, GC – gastric cancer, SDI – socio-demographic index.
